# Mutating two putative phosphorylation sites on ZHP-3 does not affect its localization or function during meiotic chromosome segregation

**DOI:** 10.17912/micropub.biology.000354

**Published:** 2021-01-18

**Authors:** Anna E. Russo, Christian R. Nelson, Needhi Bhalla

**Affiliations:** 1 Department of Molecular, Cell and Developmental Biology, University of California, Santa Cruz, Santa Cruz, CA 95064

## Abstract

Meiotic chromosome segregation depends on crossover recombination to link homologous chromosomes together and promote accurate segregation in the first meiotic division. In *Caenorhabditis elegans*, a conserved RING finger protein, ZHP-3, is essential for meiotic recombination and localizes to sites of crossover formation. Whether ZHP-3 is regulated to promote recombination remains poorly understood. *In vitro* analysis identified two putative CHK-1 kinase phosphorylation sites on ZHP-3. However, mutation of the phosphorylation sites identified *in vitro* had no effect on meiotic recombination or localization of ZHP-3. Thus, these two phosphorylation sites appear to be dispensable for ZHP-3’s role in meiotic recombination or its localization.

**Figure 1.**
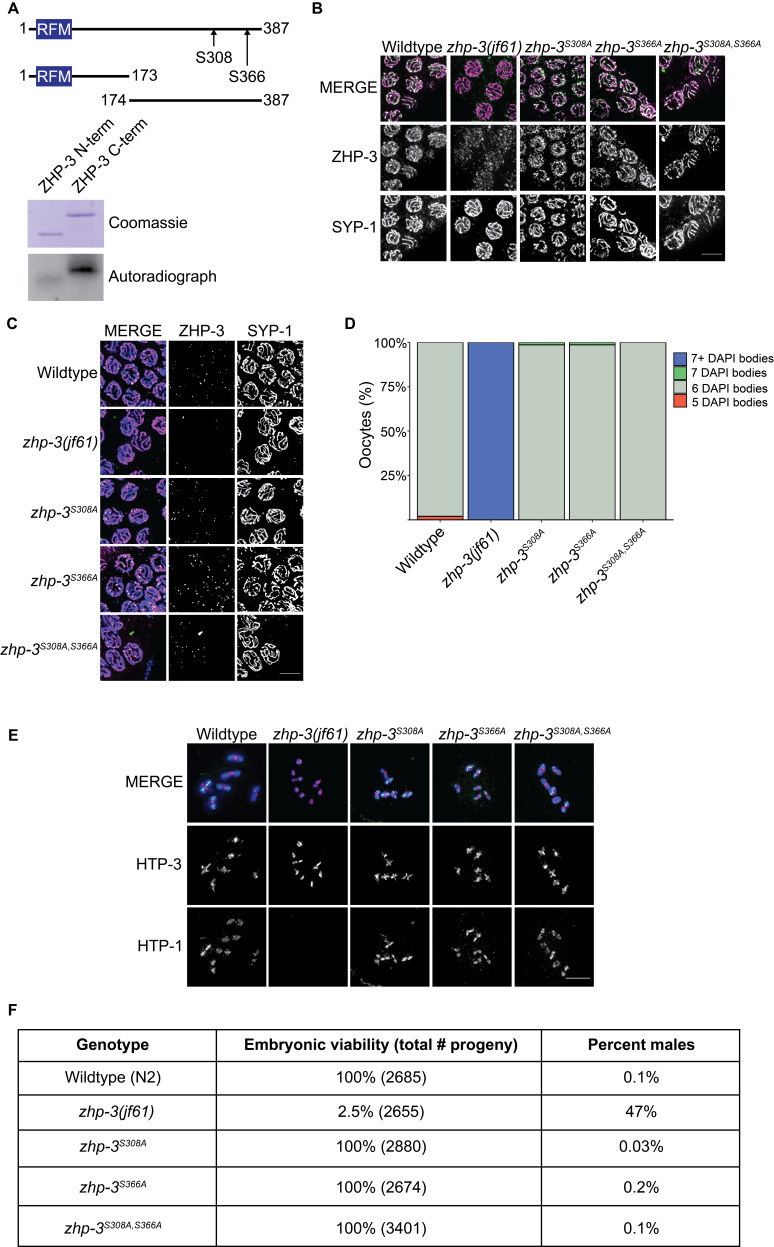
**A.** Top: Cartoon depicting the protein structure of ZHP-3 with phosphorylated residues indicated. RFM indicates the ring finger motif. Bottom: Gel stained with Coomassie blue and autoradiograph of *in vitro* kinase assays with N- and C-terminal regions of ZHP-3 and human Chk1. **B.** Mid pachytene nuclei stained with ZHP-3 (green) and SYP-1 (magenta) antibodies in wildtype germlines and *zhp-3 (jf61)* null, *zhp-3^S308A^*, *zhp-3^S366A^*, and *zhp-3^S308A, S366A^* mutant germlines. **C.** Late pachytene nuclei stained with ZHP-3 (green) and SYP-1 (magenta) antibodies in wildtype germlines and *zhp-3 (jf61)* null, *zhp-3^S308A^*, *zhp-3^S366A^*, and *zhp-3^S308A, S366A^* mutant germlines. **D.** Quantification of average number of DAPI bodies in wildtype oocytes and *zhp-3 (jf61)* null, *zhp-3^S308A^*, *zhp-3^S366A^*, and *zhp-3^S308A, S366A^* oocytes. **E.** Bivalents stained with DAPI and antibodies against HTP-3 (magenta) and HTP-1 (green) in wildtype oocytes and *zhp-3 (jf61)* null, *zhp-3^S308A^*, *zhp-3^S366A^*, and *zhp-3^S308A, S366A^* mutant oocytes. **F.** Quantification of embryonic viability and total number of male progeny in wildtype, *zhp-3 (jf61)* null, *zhp-3^S308A^*, *zhp-3^S366A^*, and *zhp-3^S308A, S366A^* strains. All scale bars indicate 5 microns. Significance was assessed using two-sided Wilcoxon-Mann-Whitney tests.

## Description

Haploid sex cells such as sperm and eggs depend on a specialized form of cell division called meiosis. Throughout prophase of meiosis, homologous chromosomes pair, synapse, and recombine to form a physical link between them called a chiasma. Chiasmata are essential for proper segregation (Page *et al.* 2003). Defects in meiotic recombination can lead to gametes that contain an incorrect number of chromosomes (aneuploidy), which can cause infertility, miscarriages, and genetic disorders such as Down Syndrome. Therefore, identifying how recombination is regulated is crucial for understanding multiple aspects of human reproductive health.

Previous research in *C. elegans* has shown that several proteins called crossover factors localize to the designated crossover site to promote recombination (Bhalla *et al.* 2008, Yokoo *et al.* 2012). One of these crossover factors, ZHP-3 (Zip homologous protein 3), is highly conserved, has a budding yeast counterpart that acts as a SUMO ligase (Cheng *et al.* 2006) and is essential for crossover formation (Jantsch *et al.* 2004). Therefore, when ZHP-3 is absent, chromosome pairs are not linked by chiasmata. Because sex determination in *C. elegans* is based on the number of X chromosomes, with hermaphrodites having two X chromosomes while males have one, a high incidence of male (HIM) phenotype is diagnostic for chromosome segregation errors. Loss of ZHP-3 results in a high rate of embryonic lethality and a HIM phenotype (Jantsch *et al.* 2004), both indicating chromosome segregation errors. We’ve shown that ZHP-3’s localization is dynamic throughout prophase: in mid-pachytene ZHP-3 is found all along the synaptonemal complex (SC) between synapsed homologous chromosomes. During late pachytene, it re-localizes and concentrates at the crossover site (Bhalla *et al.* 2008). How ZHP-3 achieves this dynamic localization to promote meiotic recombination is currently unknown.

Meiotic proteins are phosphorylated to promote essential steps in meiotic prophase, including stabilizing the SC (Nadarajan *et al.* 2017), designating the short arm of chromosome pairs to promote two step cohesion loss (Sato-Carlton *et al.* 2018), and activating negative feedback loops in response to defects in synapsis or crossover formation (Kim *et al.* 2015). CHK-1 (Checkpoint Kinase 1) localizes to early and mid-pachytene nuclei (Woglar *et al.* 2013), correlating with when ZHP-3 is present along the SC. Similar to SC proteins that are known to be phosphorylated (Nadarajan *et al.* 2017, Sato-Carlton *et al.* 2018), ZHP-3 also contains an unstructured C-terminal tail containing multiple serine residues (Reynolds *et al.* 2013). Here we test whether CHK-1 is a potential regulator of ZHP-3’s localization and if phosphorylation of two residues on ZHP-3 is required for its dynamic localization and/or to promote crossover formation.

To determine if ZHP-3 is post-translationally modified, we generated truncated versions of ZHP-3 protein, purified these from BL21 codon plus cells, and performed *in vitro* kinase assays with radiolabeled P^32^ and human Chk1 to determine if ZHP-3 is phosphorylated. We found that when ZHP-3 samples were incubated with human Chk1 and P^32^, ZHP-3 is robustly phosphorylated on its C-terminus but not its N-terminus (Figure 1A). To identify the phosphorylated residues on ZHP-3, we sent samples for mass spectrometry and identified serine 308 and serine 366 as the phosphorylated residues (Figure 1A).

To determine the significance of these phosphorylation events on ZHP-3’s function and meiotic recombination *in vivo*, we used CRISPR/Cas9 genome editing (Arribere *et al.* 2014, Paix *et al.* 2015) to mutate serines 308 and 366 to alanines (*zhp-3^S308A^* and *zhp-3^S366A^*) so that they can no longer undergo phosphorylation. Along with generating the single phosphorylation mutant strains, we also created the double phosphorylation mutant (*zhp-3^S308A, S366A^*) to test for redundancy between these two sites.

To determine if these mutations result in defects in ZHP-3’s localization, we first stained mid-pachytene nuclei with antibodies against ZHP-3 and SYP-1 to determine if ZHP-3 phosphorylation mutants can still localize along the SC. In wildtype germlines, ZHP-3 is found all along the SC and colocalizes with SYP-1 (Bhalla *et al.* 2008, Figure 1B). We found that ZHP-3^S308A^, ZHP-3^S366A^ and ZHP-3^S308A, S366A ^colocalized with SYP-1 on the SC (Figure 1B), similar to wildtype ZHP-3, indicating that these mutations do not affect ZHP-3’s ability to localize to the SC.

To determine if ZHP-3’s localization is affected in late pachytene, we tested whether ZHP-3^S308A^, ZHP-3^S366A^, and ZHP-3^S308A, S366A^ can still properly localize to crossovers. We found that ZHP-3^S308A^, ZHP-3^S366A^, and ZHP-3^S308A, S366A^ localized in distinct foci on chromosomes (Figure 1C), similar to wildtype ZHP-3, indicating that the single and double phosphorylation mutations do not affect ZHP-3’s ability to localize to crossovers.

To determine if mutation of these phosphorylation sites results in defects in recombination or chromosome morphology, we quantified the number of DAPI stained bodies in oocytes. Wildtype oocytes contain 6 DAPI stained bodies, corresponding to 6 chromosome pairs (bivalents) held together by chiasmata (Dernburg *et al.* 1998). Recombination is lost in *zhp-3(jf61)* null mutants, resulting in 12 DAPI stained bodies corresponding to non-recombinant univalents (Jantsch *et al.* 2004, Figure 1D). In *zhp-3^S308A^*, *zhp-3^S366A^*, and *zhp-3^S308A, S366A ^*mutants, oocytes had an average of 6 DAPI bodies (Figure 1D).

We also stained germlines with antibodies against HTP-3 and HTP-1, meiotic chromosomal proteins that preferentially localize to the long and short arms of bivalents (Martinez-Perez *et al.* 2008) to determine if there were defects in bivalent morphology. HTP-3 localizes to both the long and short arm of bivalents in a cruciform pattern, and HTP-1 localizes to the long arm on wildtype bivalents (Figure 1E, Martinez-Perez *et al.* 2008). In contrast, HTP-3 and HTP-1 are lost on the long arm in *zhp-3(jf61)* null mutants (Figure 1E). We found that HTP-3 localizes to both the long and short arm and HTP-1 localizes to the long arm properly in *zhp-3^S308A^*, *zhp-3^S366A^*, and *zhp-3^S308A, S366A ^*mutants (Figure 1E), indicating that bivalent morphology is unaffected in *zhp-3* phosphorylation mutants.

Finally, we tested whether mutation of these sites results in chromosome missegregation by assessing the number of viable and male progeny for each phosphorylation mutant strain. Males are found in ~1/1000 progeny in wildtype strains, while *zhp-3(jf61)* null mutants have reported 1.3% viable progeny and ~30% males (Bhalla *et al.* 2008). We found that both the single phosphorylation mutants and double phosphorylation mutants had 100% viable progeny and 0.03%, 0.2%, and 0.1% male progeny respectively (Figure 1F), illustrating that mutation of these sites does not cause general defects in meiosis.

Regulation of meiotic recombination is essential for proper development of gametes and preventing aneuploidy. While ZHP-3 is essential for meiotic recombination in *C. elegans*, whether and how it is regulated to promote recombination remains an active area of investigation. While we found that ZHP-3 is phosphorylated on its C-terminus at S308 and S366 *in vitro*, mutation of these phosphorylation sites *in vivo* does not result in defects in ZHP-3’s localization (Figures 1B and 1C), bivalent formation (Figures 1D and 1E), or chromosome segregation (Figure 1F).

We conclude that ZHP-3 is either not regulated by these phosphorylation sites or that there is potential redundancy for ZHP-3 regulation by phosphorylation. Whether ZHP-3 is modified at other residues by phosphorylation or other post-translational modifications remain open questions in the field. It has also been recently discovered that there are additional ZHP proteins that play a role in crossover formation in *C. elegans* (Nguyen *et al.* 2018, Zhang *et al.* 2018). How these proteins coordinate to promote recombination and whether they require protein modifications are interesting questions for determining how crossover formation is regulated to promote meiotic chromosome segregation.

## Methods

***C. elegans* strains and genetics:**

The Bristol N2 *C. elegans* strain (Brenner *et al.* 1974) was used as the wildtype control for all experiments and as the parent strain for the single phosphorylation mutant CRISPR/Cas9 genome editing experiments. The *zhp-3 ^S366A^* strain was used as the background to create the *zhp-3 ^S308A ,S366A^* double phosphorylation mutant.

Strains were maintained at 20°C under standard conditions for all immunostaining experiments and maintained at 15°C for all CRISPR/Cas9 genome editing experiments.

**ZHP-3 protein expression**

Two fragments of ZHP-3, the N-terminus (aa 1-173) and the C-terminus (aa 174-387), were first amplified from cDNA, and then inserted into pDONR221 via BP reaction and shuttled into pDEST15, which contains an N-terminal GST tag, via LR reactions using the Gateway Cloning System (Invitrogen). GST-tagged ZHP-3 fragments were expressed in BL21 codon plus cells (Agilent) overnight at 18ºC (14-16 hours) to maintain solubility after induction with 0.2mM IPTG. Cells were lysed in a coffee grinder, resuspended in cold lysis buffer (1X PBS, 0.5% Tween-20, 1M NaCl, 10mM DTT) with protease inhibitor (1mM PMSF), sonicated, and then spun for 1 hour at 35k to clarify lysate. A glutathione agarose column (Sigma) was equilibrated in lysis buffer, the lysate was loaded, the column washed (1X PBS, 0.05% Tween-20, 0.5mM DTT, 0.25M KCl), washed a second time in the same buffer emitting detergent (Tween-20). Protein then was eluted from the column (50mM Tris pH8, 0.25M KCl, 5mM reduced glutathione) in 1mL fractions. The fractions containing protein (Bradford Assay, Bio-Rad) were combined and dialyzed overnight (50mM Hepes-KOH pH7.4, 0.25M KCl, 30% glycerol) and then aliquoted and stored at -80 ºC.

***In vitro* Kinase Assays**

Kinase assays were performed at 30ºC in 20uL reactions with 1X kinase buffer (50mM Tris pH7.4, 1mM DTT, 25mM beta-glycerophosphate, 5mM MgCl2 ,10mM ATP) for 30 minutes. Reactions included 0.5uL ^32^P- γ-ATP (Perkin Elmer), 0.5uL of activated Chk1 (Sigma), and 2ug of substrate. Reactions were quenched by adding 20uL 2X sample buffer and boiling for 5 minutes. Reactions were run out in duplicate on two 10% SDS-PAGE gels, one was stained with Coomassie to verify equivalent amounts of substrate were utilized, and one was dried and then exposed to a phosphor screen (GE Life Sciences) for analysis of phosphorylation.

**Mass Spectrometric Analysis**

*In vitro* assays were performed as above without radiolabeled ATP. Samples were frozen in liquid nitrogen and sent to the QB3/Chemistry Mass Spectrometry Facility at University of California, Berkeley, which performed the desalting and trypsin digest prior to mass spectrometry. Mass spectrometry produced multiple peptides of the protein sequence of the C-terminus and GST tag, indicating good coverage, and identified S308 and S366 as the *in vitro* phosphorylated residues.

**Generating phosphorylation mutant strains using CRISPR/Cas9 genome editing:**

The *zhp-3^S308A^, zhp-3^S366A^,* and *zhp-3^S308A, S366A^* phosphorylation mutant strains were all generated using a CRISPR/Cas9 ribonucleoprotein (RNP) approach (Paix *et al.* 2015) combined with the coconversion genome editing method (Arribere *et al.* 2014).

N2 young adults were injected with a mix that included a *zhp-3* specific crRNA (IDT-Alt-R^TM^, 100μM stock), the *dpy-10* coconversion crRNA (IDT-Alt-R^TM^, 100μM stock) , tracrRNA (IDT-Alt-R^TM^, 100μM stock) a *zhp-3* specific repair oligo (IDT, 50μM stock) the *dpy-10* coconversion repair oligo (IDT, 50μM stock), and purified Cas9 protein (40μM stock). F1 *roller* and *dpy* progeny from injected P0’s were isolated to individual plates, and non-rolling non *dpy* siblings from jackpot plates (> 10 transformed worms) were grouped 3-5 F1’s per plate. After F2 progeny were generated, F1’s were screened for the respective phosphorylation mutant allele via PCR and restriction digest. F2’s from a F1 plate that had the confirmed mutant allele were singled to identify homozygotes for each phosphorylation mutation. All phosphorylation mutant strains were confirmed via sequencing. Phosphorylation mutant strains were outcrossed to N2 worms 1-2 times prior to analysis.

For creating the *zhp-3^S308A^* strain, the following sgRNA was used: 5’-GGA CAA AAC UAG UAU GUC ACG UUU UAG AGC UAU rGrCrU-3’. The following single stranded oligo was used for the S308A mutation repair template: 5’-GGA CAA AAC TAG TAT GTC ACT CGA AAA TTG GAG GCA AAA TAG AGC GAA TGC ATT CGG AGT GCA TGA TAT GTG AGA TAT TTT CAA AGT ATC TGT GAT TCC TAA TTC-3’.

For creating the *zhp-3^S366A^* strain, the following sgRNA was used: 5’– AAA AGA GUG AAU GAC AGA CCG UUU UAG AGC UAU rGrCrU –3’. The following single stranded oligo was used for the S366A mutation repair template: 5′ – GTG CAG CTG GAT TCG ACC GTC AGC AGA TAC AAG AAA TGC GTC GAA TCT CAG CTC AAC CTG GTC TGT CAC CCA CTC TTT TAT TCC TCA ATT TCC CTC GAC CCT CTC ATC –3’.

For creating the *zhp-3^S308A, S366A^* strain, the same S308A sgRNA and oligo repair template above were used to create the S308A mutation in the *zhp-3^S366A^* mutant background.

**DAPI staining and Immunostaining:**

Adult hermaphrodites were fixed and stained 24-26 hours post L4 larval stage as in (Bhalla *et al.* 2005). For visualizing bivalents in mature oocytes for quantification, adult hermaphrodites were fixed and stained 48 hours post L4 stage.

For assessing ZHP-3 localization, the following primary antibodies were used: guinea pig anti-ZHP-3 (1:250) (Bhalla *et al.* 2008), rabbit anti-SYP-1 (1:250) (MacQueen *et al.* 2002), chicken anti-HTP-3 (1:500) (MacQueen *et al.* 2005). All secondary antibodies were used at a 1:500 dilution and included: Alexa 488 anti guinea pig (Invitrogen), Cy3 anti rabbit (Jackson Immunochemicals), and Cy5 anti chicken (Jackson Immunochemicals).

For assessing bivalent formation, the following primary antibodies were used: rabbit anti-HTP-1 (1:400) (Martinez-Perez *et al.* 2008) and chicken anti-HTP-3 (1:500) (MacQueen *et al.* 2005). All secondary antibodies were used at a 1:500 dilution and included: Alexa 488 anti rabbit (Invitrogen) and Cy3 anti chicken (Jackson Immunochemicals).

DAPI in 1X PBST was used at a concentration of 1:10,000 for all experiments.

Images of immunostaining experiments were obtained using a DeltaVision Personal DV system (Applied Precision) equipped with a 100× N.A. 1.40 oil-immersion objective (Olympus), resulting in an effective XY pixel spacing of 0.064 or 0.040 μm. Z-stacks were collected at 0.2-μm *Z*-spacing and processed by constrained, iterative deconvolution. Imaging, image scaling, and analysis were performed using functions in the softWoRx software package. Projections were calculated using a maximum intensity algorithm. Composite images from immunostaining experiments were processed and some false coloring was performed using Fiji.

**Quantification of bivalents:**

Bivalent quantification was performed on worms 48 hours post L4 larval stage. Each genotype included at least 3 germlines for quantification.

**Viability assay and male counts:**

N2, *zhp-3(jf61)*,*zhp-3^S308A^, zhp-3^S366A^,* and *zhp-3^S308A, S366A ^*late L4 stage worms were isolated to plates and maintained at 20 degrees. F1 embryos and oocytes were scored at 24, 48 and 72 hours post L4 larval stage. Once F1’s reached the late L4 stage, viable and male progeny were scored. The total brood size, percent viability and percent HIM phenotype were calculated for each strain. A two-sided Wilcoxon-Mann-Whitney test was performed to assess statistical significance.

**Figures and statistics:**

Figures were assembled using Adobe Illustrator. All histograms and statistics were performed using R (R core team) with the ggplot2 package (Wickham *et al.* 2016). For bivalent quantification, viability assay and male counts, a two-sided Wilcoxon-Mann-Whitney test was used to determine statistical significance.

## Reagents

**Table:** 

Strain	Genotype	Shorthand Name	Available from
Bristol N2	*Caenorhabditis elegans*	Wildtype	CGC
UV1	*zhp-3(jf61) I/hT2[bli-4(e937) let-?(q782) qIs48 (I:III)]; + III/hT2[bli-4(e937) let-?(q782) qIs48 (I:III)]*	*zhp-3(jf61)* null	CGC
BHL966	*zhp-3(blt6)*	*zhp-3^S308A^*	Bhalla lab
BHL967	*zhp-3(blt7)*	*zhp-3^S366A^*	Bhalla lab
BHL968	*zhp-3(blt8)*	*zhp-3^S308A, S366A^*	Bhalla lab

**Table:** 

Antibody	Animal and clonality	Description
anti-ZHP-3	Guinea pig polyclonal	Source: Dernburg lab (Bhalla *et al.* 2008)
anti-SYP-1	Rabbit polyclonal	Source: Villeneuve lab (MacQueen *et al.* 2002)
anti-HTP-1	Rabbit polyclonal	Source: Villeneuve lab (Martinez-Perez *et al.* 2008)
anti-HTP-3	Chicken polyclonal	Source: Dernburg lab (MacQueen *et al.* 2005)

**Table:** 

Reagent	Sequence	Description
*zhp3^S308A ^sgRNA*	5’-GGA CAA AAC UAG UAU GUC ACG UUU UAG AGC UAU rGrCrU-3’	sgRNA used to create the *zhp3^S308A ^mutation.* Synthesized from IDT.
*zhp3^S366A ^sgRNA*	5’– AAA AGA GUG AAU GAC AGA CCG UUU UAG AGC UAU rGrCrU –3’	sgRNA used to create the *zhp3^S366A ^mutation.* Synthesized from IDT.
*zhp3^S308A ^oligo repair template*	5’-GGA CAA AAC TAG TAT GTC ACT CGA AAA TTG GAG GCA AAA TAG AGC GAA TGC ATT CGG AGT GCA TGA TAT GTG AGA TAT TTT CAA AGT ATC TGT GAT TCC TAA TTC-3’	Single stranded oligo repair template used to create the *zhp3^S308A ^mutation.* Synthesized from IDT.
*zhp3^S366A ^oligo repair template*	5′ – GTG CAG CTG GAT TCG ACC GTC AGC AGA TAC AAG AAA TGC GTC GAA TCT CAG CTC AAC CTG GTC TGT CAC CCA CTC TTT TAT TCC TCA ATT TCC CTC GAC CCT CTC ATC –3’	Single stranded oligo repair template used to create the *zhp3^S366A ^mutation.* Synthesized from IDT.
*dpy-10* sgRNA	5′ – GCU ACC AUA GGC ACC ACG AGG UUU UAG AGC UAU rGrCrU – 3′	sgRNA used to create the *dpy-10* mutation. Sequence sourced from Arribere *et al.* 2014. Synthesized from IDT.
*dpy-10* oligo repair template	5′ – CACTTGAACTTCAATACGGCAAGATGAGAATGACTGGAAACCGTACCGCATGCGGTGCCTATGG TAGCGGAGCTTCACATGGCTTCAGACCAACAGCCTAT – 3′	Single stranded oligo repair template used to create the *dpy-10* mutation. Sequence sourced from Arribere *et al.* 2014. Synthesized from IDT.
